# Angiogenic Biomarkers: Are They Good Tools to Predict Perinatal Outcomes in Hypertensive Disorders of Pregnancy? A Retrospective Cohort Study

**DOI:** 10.3390/diagnostics15070799

**Published:** 2025-03-21

**Authors:** Magdalena Bednarek-Jędrzejek, Katarzyna Maksym, Stepan Feduniw, Slagjana Simeonova Krstevska, Igor Samardziski, Tomasz Góra, Michał Ciebiera, Adrianna Zahorowska, Katarzyna Pruś, Sylwia Dzidek, Hanna Jasiak-Jóźwik, Martyna Drzycimska, Ewa Kwiatkowska, Andrzej Torbé, Sebastian Kwiatkowski

**Affiliations:** 1 Department of Gynecology and Obstetrics, Pomeranian Medical University, 70-111 Szczecin, Poland; adzahorowska@gmail.com (A.Z.); katarzynakasiap04@gmail.com (K.P.); sylwiadzidek@wp.pl (S.D.); hania.jasiak@gmail.com (H.J.-J.); martynadrzycimska@gmail.com (M.D.); torbea@wp.pl (A.T.); kwiatkowskiseba@gmail.com (S.K.); 2 EGA Institute for Women’s Health, University College London, London WC1E 7HB, UK; k.maksym@ucl.al.uk; 3 Fetal Medicine Unit, University College London Hospitals NHS Trust, London W1T 7DN, UK; 4 Department of Gynecology, University Hospital of Zurich, 8091 Zurich, Switzerland; stepan.feduniw@gmail.com; 5 Medical Faculty, University Clinic for Gynecology and Obstetrics, 1000 Skopje, North Macedonia; simeonovas@yahoo.com (S.S.K.); igidoc@yahoo.com (I.S.); 6 Clinical Department of Gynecology and Obstetrics, Municipal Hospital, John Paul II, 35-241 Rzeszów, Poland; minddin@gmail.com; 7 Second Department of Obstetrics and Gynecology, Center of Postgraduate Medical Education, 00-189 Warsaw, Poland; 8 Warsaw Institute of Women’s Health, 00-189 Warsaw, Poland; 9 Department of Nephrology, Transplantology and Internal Medicine, Pomeranian Medical University, 70-111 Szczecin, Poland; ewakwiat@gmail.com

**Keywords:** preeclampsia, angiogenic biomarkers, perinatal outcomes, time to delivery, sFlt-1, PlGF

## Abstract

**Background**: The sFlt-1/PlGF ratio has proven predictive value in diagnosing preeclampsia. Referring to a study from 18 American perinatal centers, we present results from 2 European centers showing the significant value of those markers in predicting severe perinatal outcomes in hypertensive disorders of pregnancy. **Methods**: A total of 1630 patients with suspected or confirmed placental insufficiency, hospitalized in two tertiary perinatal centers in Poland and Macedonia, were assessed for their sFlt-1/PlGF ratio. Due to incomplete data, perinatal outcomes were only obtained for 1196 patients. They were sorted into two groups according to the value of the sFlt-1/PlGF ratio (<40 and ≥40). The aim of this study was to predict adverse perinatal outcomes in terms of days to delivery, gestational age, birth weight, and cord blood pH. **Results**: The strongest negative correlation was observed between the index values and the number of days until delivery (R = −0.48; *p* < 0.001). In a group of patients with an index value of ≥40, the AUC was 0.9955 (95% CI: 0.9913 to 0.9996), with a sensitivity of 52%, a specificity of 78%, a positive predictive value of 77%, and a negative predictive value of 53%. For patients who were tested before 37 weeks of gestation, 66% of women with a ratio of ≥40 delivered within 7 days of the test, and 80% of those with a ratio of <40 delivered more than 7 days after the test, with a sensitivity of 68%, a specificity of 79%, a positive predictive value of 66%, and a negative predictive value of 80%. **Conclusions**: In women with hypertensive disorders, the sFlt-1/PlGF ratio can be used to predict the time to delivery. A cut-off of 40 is very useful in predicting severe perinatal outcomes.

## 1. Introduction

Preeclampsia (PE) is a multisystem disease that typically affects 2–5% of pregnant women and is one of the leading causes of maternal and perinatal morbidity and mortality [1]. It is a hypertensive disorder that is related to 2–8% of pregnancy-related complications worldwide [2]. Women who survive preeclampsia have reduced life expectancy, with increased risks of stroke, cardiovascular disease, and diabetes, while babies born from a preeclamptic pregnancy have increased risks of preterm birth, perinatal death, neurodevelopmental disability, and cardiovascular and metabolic disease later in life [3]. The best treatment for severe preeclampsia is still immediate delivery, which is why the appropriate time of diagnosis and the choice of the moment of childbirth are so important. It is vital to select the moment when the baby will be exposed to the fewest possible complications and the mother will be protected from complications related to preeclampsia.

Around 10 years ago, authors focused on the appropriate way to diagnose preeclampsia, and the effectiveness of the sFlt-1/PlGF ratio in predicting preeclampsia was confirmed [4,5,6,7,8,9,10]. We know how to predict preeclampsia, how to reduce the risk in high-risk populations, and how to treat it, but the question remains as to how we can avoid problems after PE. Currently, authors are increasingly focusing on the possibility of using various models to predict preeclampsia complications and poor perinatal outcomes [11,12,13]. Many publications present the role of markers of impaired angiogenesis in predicting perinatal outcomes [14,15,16,17,18].

In our previous publication, we demonstrated the usefulness of the sFlt-1/PlGF ratio in the appropriate ranges for the prediction of preeclampsia in predicting poor perinatal outcomes, such as preterm birth and associated prematurity, SGA (small for gestational age) fetuses, pH values below 7.1 obtained from the umbilical artery shortly after birth, and newborns with low Apgar scores [19]. Two years ago, a large American study involving 18 perinatal centers showed that the measurement of serum sFlt-1/PlGF helped stratify the risk of progressing to severe preeclampsia (sPE) within the coming fortnight [20]. Here, referring to a study conducted in 18 American perinatal centers, we present results from 2 European centers showing the significant value of markers of impaired angiogenesis in predicting severe perinatal outcomes in hypertensive disorders of pregnancy.

## 2. Materials and Methods

The study group consisted of 1630 pregnant patients between 18 and 41 weeks’ gestation with suspected or confirmed placental insufficiency hospitalized in two tertiary perinatal centers in Poland and Macedonia: the Clinical Department of Obstetrics and Gynaecology, Pomeranian Medical University, Szczecin, Poland; and the University Clinic of Gynaecology and Obstetrics, Skopje, Republic of Macedonia. The diagnostic criteria for individual disease entities, in line with the applicable guidelines, were used to qualify the patients for inclusion in this study (Table 1).

Once admitted, the patients were managed using local protocols developed in line with widely accepted guidelines [21]. The levels of the angiogenesis markers sFlt-1 and PlGF were measured in maternal serum for all participants after informed consent was obtained. The blood for the tests was taken on enrolment. The results were given after 30 min. The sFlt-1 and PlGF levels were measured using Elecsys sFlt-1 and Elecsys PLGF immunoassays, respectively.

### Statistical Analyses

The results were analyzed using GraphPad Prism version 10.2.3 (GraphPad Software, Boston, MA, USA; www.graphpad.com). The normality of the data was assessed using the D’Agostino–Pearson test, Anderson–Darling test, Shapiro–Wilk test, and Kolmogorov–Smirnov test. The statistical analysis included Spearman correlation, simple logistic regression, and linear regression, along with the Mann–Whitney, Kruskal–Wallis, and chi-squared tests. This study was carried out with approval from Pomeranian Medical University’s institutional review board (no. n.KB-0012/122/12).The patients were divided into two groups according to the sFlt-1/PlGF index value (<40 and ≥40). Subsequently, perinatal outcomes were compared between these two groups, particularly the number of days from sFlt-1 and PlGF determination to delivery, cord blood pH, neonatal birth weight, duration of gestation at the time of ratio determination, and gestational age at birth.

## 3. Results

A cumulative total of 1630 patients were included in the analysis. The full data were available for 1196 patients. The baseline characteristics of the entire study population are provided in Table 2. In total, 53% of patients were primiparas. The median gestational age at enrolment was 38 weeks (min: 18; max: 42; 95% CI: 38). All of the patients were Caucasian. The study population was representative of the population of pregnant women with some forms of placental insufficiency in Eastern Europe. The median enrolment blood pressures were 140 mm Hg systolic (min 90; max 223; 95% CI: 138; 140) and 89 mm Hg diastolic (min 50; max 140; 95% CI: 88; 90), with a median sFlt-1/PlGF ratio of 28/16 (min 0.38; max 1645; 95% CI: 2.978 to 204.8).

In the whole group of patients, 30% developed preeclampsia. Among the delivered babies, 21% were SGA. In the entire study group, we found a negative correlation between the index values and the perinatal outcomes (Table 3). The strongest negative correlation was found between the index values and the number of days until delivery (R= −0.48; *p* < 0.001) (95% CI: −0.5288 to −0.4395). There were strong negative correlations between the ratio and birth weight (R= −0.36; *p* < 0.001) (95% CI: −0.4133 to −0.3117) and between the ratio and gestational age (R= −0.35; *p* < 0.001) (95% CI: −0.3991 to −0.2966).

**Figure 1:**
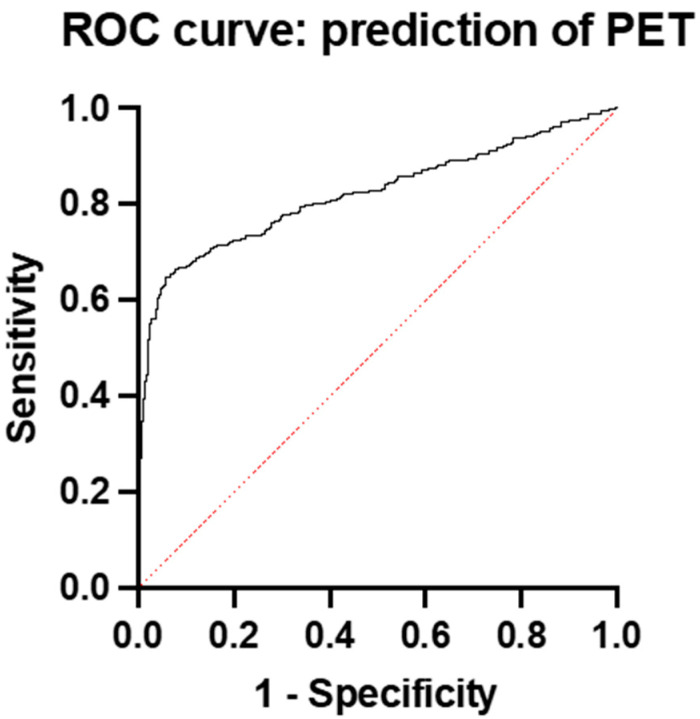
ROC curve and AUC for prediction of preeclampsia with sFlt-1/PlGF ratio in whole group.

The predictive performance of the sFlt-1/PlGF ratio in predicting PE was quite high. The AUC of the ratio was 0.82 (95% CI: 0.7920 to 0.8519) (Figure 1).

**Figure 2:**
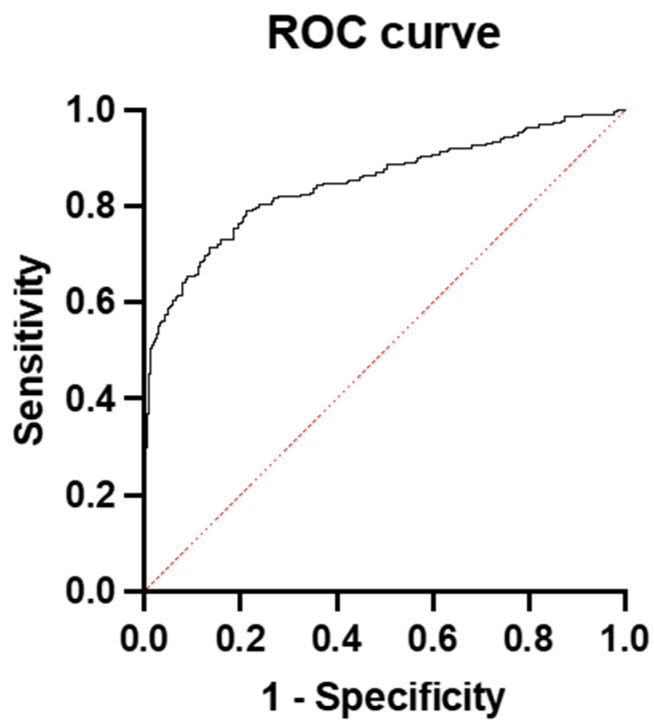
ROC curve and AUC for the prediction of preeclampsia with the sFlt-1/PlGF ratio and systolic blood pressure in the whole group.

We also ran a multiple logistic regression model to predict PE using BMI, BP, and the sFlt-1/PLGF ratio. Only systolic BP and the ratio were predictive, and they were not much better than the ratio alone. The AUC of the sysBP and ratio was 0.85 (95% CI: 0.8149 to 0.8819) (Figure 2).

**Figure 3:**
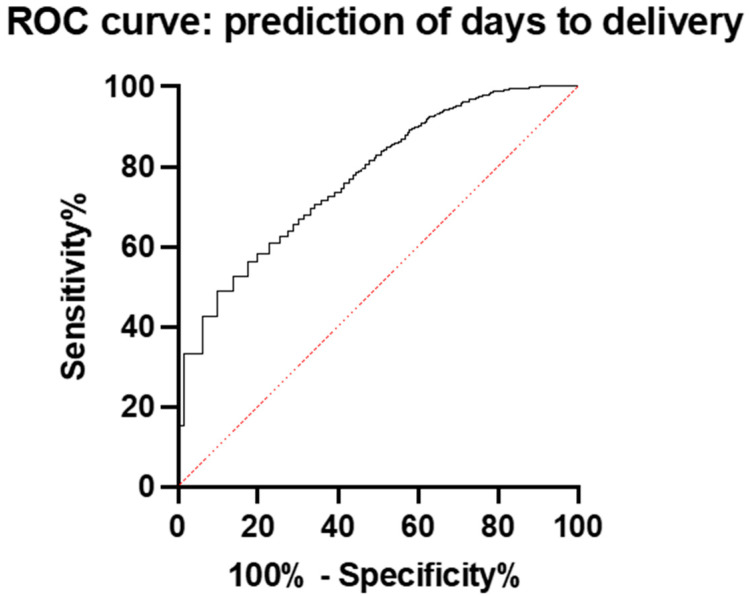
ROC curve and AUC for the prediction of days to delivery with the sFlt-1/PlGF ratio in the whole group.

In the entire study group, the prediction of perinatal outcomes was the best with days to delivery. The predictive value of the sFlt-1/PlGF ratio for days to delivery was quite good; the AUC was 0.77 (95% CI: 0.7544 to 0.7907) (Figure 3).

Based on the American publication, we divided our patients into two groups based on sFlt-1/PlGF values of ≤40 and >40 (Table 4).

**Figure 4:**
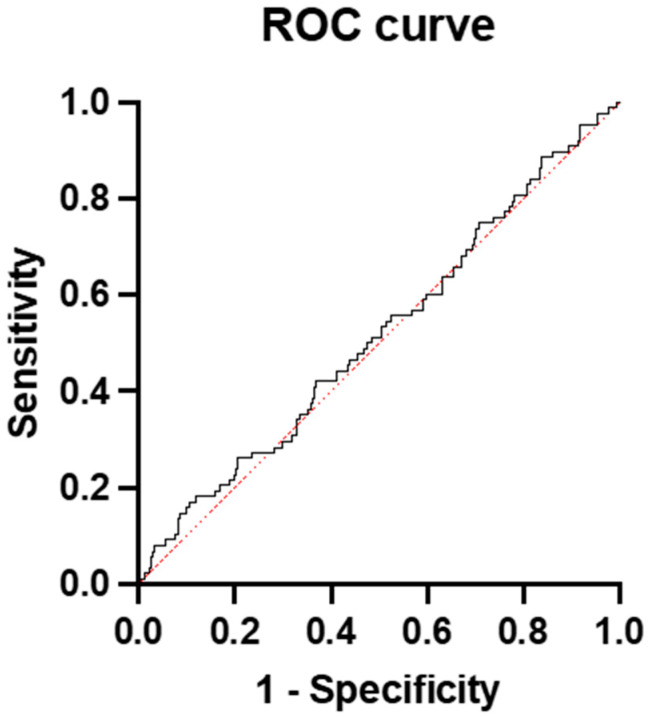
ROC curve and AUC for the prediction of preeclampsia with the sFlt-1/PlGF ratio in the group with a ratio of ≤40.

In the group with a ratio of ≤40, only 12.7% developed preeclampsia. The ratio had no predictive value for PE in this group; the AUC was 0.52 (95% CI: 0.4515 to 0.5826) (Figure 4).

**Figure 5:**
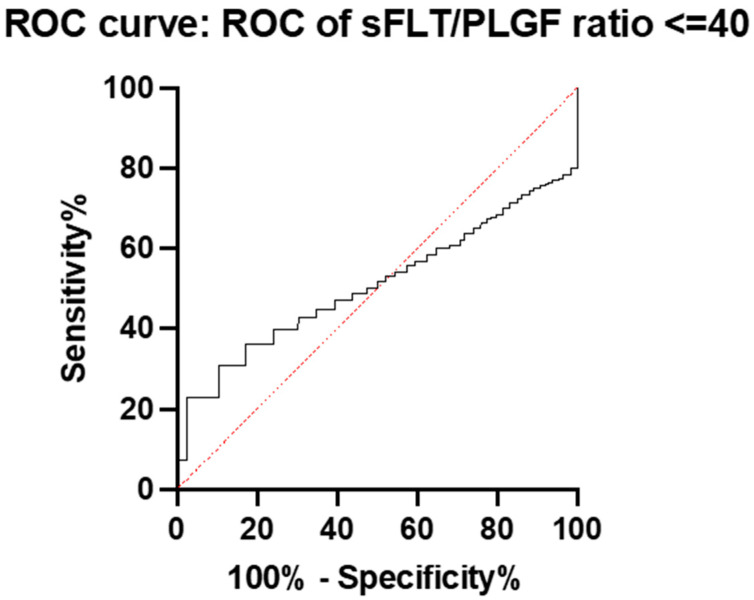
ROC curve and AUC for the prediction of days to delivery with the sFlt-1/PlGF ratio in the group with a ratio of ≤40.

In the group with a small ratio, there were 120 SGA babies (23.7%); their median birth weight was 3000 g (95% CI: 2903 to 3007), and the median birth weight centile was 38.01 (95% CI: 39.98 to 44.42). In the group with the higher ratio, there were 130 SGA babies (18.9%); their median birth weight was 2500 g (95% CI: 2333 to 2489), and the median birth weight was 24.67 (95% CI: 30.57 to 35.45). The most important difference that we found between the groups was the value of the ratio for predicting days to delivery. In the group with a ratio of ≤40, the ratio had no statistically significant predictive value; the AUC was 0.5081 (95% CI: 0.4764 to 0.5399) (Figure 5).

**Figure 6:**
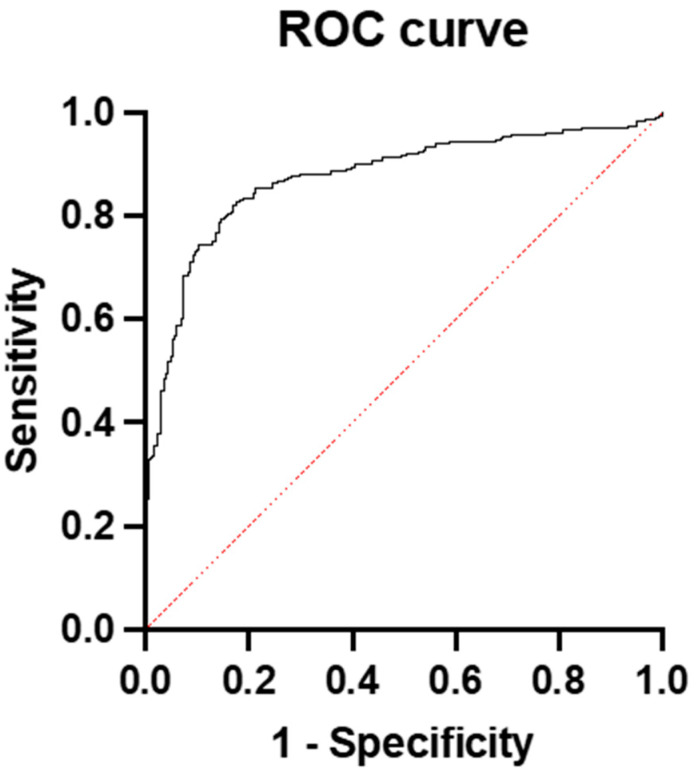
ROC curve and AUC for the prediction of preeclampsia with the sFlt-1/PlGF ratio in the group with a ratio of >40.

The cutoff of 40 showed good predictive value of the ratio for predicting preeclampsia; the AUC was 0.8738 (95% CI: 0.8423 to 0.9053). In this group, 55% of patients developed preeclampsia (Figure 6).

**Figure 7:**
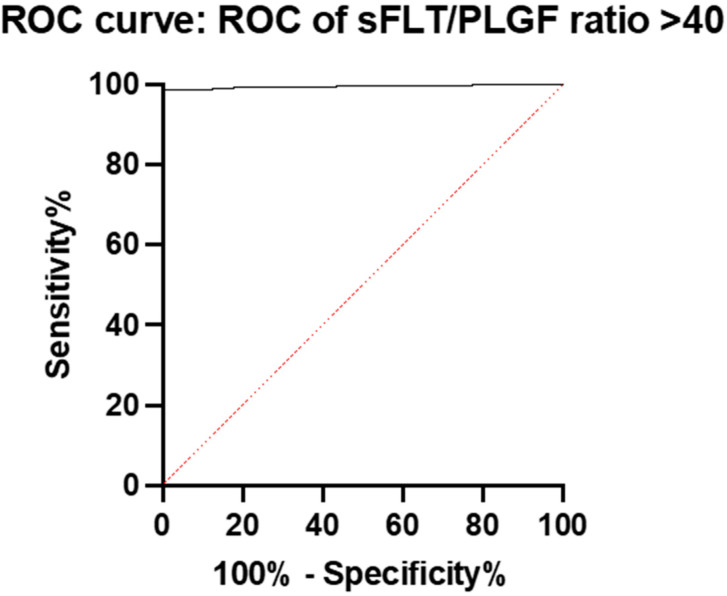
ROC curve and AUC for the prediction of days to delivery with the sFlt-1/PlGF ratio in the group with a ratio of >40.

We found that the best predictive value of the ratio to predict days to delivery was in the group with a ratio of >40. The AUC was 0.9955 (95% CI: 0.9913 to 0.9996), with a sensitivity of 52%, a specificity of 78%, a positive predictive value of 77%, and a negative predictive value of 53% (Figure 7).

We found that gestational age was a confounding factor, so we decided to measure the days to delivery in both preterm and term delivery. For patients who were tested before 37 weeks of gestation, 66% of women with a ratio of ≥40 delivered within 7 days of the test, and 80% of those with a ratio of <40 delivered more than 7 days after the test, with a sensitivity of 68%, a specificity of 79%, a positive predictive value of 66%, and a negative predictive value of 80%. For patients tested at 37 weeks or later, 90% of patients with a ratio of ≥40 delivered within 7 days, as did 83% with a ratio of <40, with a sensitivity of 44%, a specificity of 73%, a positive predictive value of 90%, and a negative predictive value of 17%.

## 4. Discussion

The PROGNOSIS study proved the utility of the sFlt-1/PlGF index in diagnosing and excluding preeclampsia, depending on its value [8]. High index values (>85) also indicate a shorter time from diagnosis to delivery. This publication had a major influence on other researchers and clinicians in the diagnosis of preeclampsia. Appropriate index ranges of <38, 38–85, and >85 are used in many places on a daily basis to diagnose and exclude preeclampsia. More and more researchers are focusing on the use of markers not only in the diagnosis of preeclampsia but also in predicting poor perinatal outcomes. Some studies have focused on the long-term effects on the mother after preeclampsia [22,23]. Others, however, are looking for solutions to predict poor perinatal outcomes in pregnancies with suspected placental insufficiency.

In our previous publication, we assessed the perinatal outcomes of pregnancies with suspected placental insufficiency according to the sFlt-1/PlGF ratio ranges recommended by the PROGNOSIS study [19]. Currently, more and more researchers are assessing how perinatal outcomes vary depending on the population. Patients from Sweden and China [24] have been compared, as well as studies performed only on the Indian population, or among patients from New Zealand [25,26]. The PROGNOSIS study itself was conducted on patients from Western Europe.

In this study, we used a cut-off point of 40, as in the US population [20]. Our findings showed that, as in the cited study, index values of >40 were indicative of patients with a higher risk of developing preeclampsia. As many as 55% of the >40 group developed symptoms of preeclampsia, compared to only 12.7% in the ≤40 group. As in our previous publication, the values of the sFlt-1/PlGF ratio showed statistical significance in relation to perinatal outcomes in the entire study population. The higher the values of markers of impaired angiogenesis, the shorter the duration of pregnancy, the lower the fetal birth weight, the lower the pH of the umbilical cord blood, and the shorter the time from determining the marker values to the day of delivery. However, we were surprised by the results after dividing our patients into groups according to the cut-off point of the index 40. The parameter that turned out to be the best predictor was the number of days until delivery from the determination of the angiogenesis markers. Among patients in the ≤40 group, this value was very low and statistically insignificant; the AUC was 0.5081 (95% CI: 0.4764 to 0.5399). However, in the >40 group, this value was extremely high and statistically significant; the AUC was 0.9955 (95% CI: 0.9913 to 0.9996).

There are studies that used only PLGF in predicting preeclampsia and time to delivery [27,28,29]. However, the sFlt-1/PlGf index was shown to be more specific in diagnosing poor perinatal outcomes [30] (PMID: 27028698). Many authors have demonstrated worse perinatal outcomes with higher index values—primarily lower fetal weight and shorter pregnancy duration [31,32,33]; however, in these works, values of 38 or 85 were considered as the cut-off point for the index values.

Recently, a publication stated that the sFlt-1/PlGF ratio is a marker of premature delivery within seven days [34]; the authors of this study used a cut-off index value of 12, with an AUC of 0.72. Our results, with a cut-off of 40, demonstrated a more accurate predictive value for days to delivery, with a median of 9 and an AUC of 0.99. However, the former study considered a group of patients with full-term pregnancies. We noticed that the gestational age was the confounding factor in our study. It is definitely easier to make the decision to give birth among patients with a full-term pregnancy. Therefore, we divided our patients into groups in which the tests were performed before and after the 37th week of pregnancy. Among patients with a non-term pregnancy, as many as 66% of patients with an index value of ≥40 gave birth within 7 days, and as many as 80% of patients with an index value of <40 gave birth more than 7 days after the determination of angiogenesis markers, with a sensitivity of 68%, a specificity of 79%, a positive predictive value of 66%, and a negative predictive value of 80%. This allows us to conclude that an index value of 40 among patients with a non-term pregnancy and suspected placental insufficiency can rule out premature delivery within 7 days. Our goal is not to end the pregnancy but to deliver the baby at the most convenient time—whether by cesarean section or by natural means—to minimize the risk of maternal or fetal complications.

### Strengths and Limitations

However, our study investigated a much larger group of patients, similar to the group of patients from the American study [20]. Both in the American population—where the study was also conducted among patients with non-term pregnancies—and in our patients from Eastern Europe, we can see how accurately the cut-off point of 40 allows for predicting a faster time to delivery among patients with an index value of >40. The group of patients with unfulfilled pregnancies requires careful analysis, which we will aim to provide in subsequent publications.

## 5. Conclusions

Women with a pregnancy complicated by placental insufficiency (with or without hypertensive manifestation) diagnosed between 18 and 41 weeks are at high risk of developing PE. The use of angiogenic biomarkers can provide a good estimation of the interval between diagnosis and delivery. The cut-off of 40 is very useful in predicting the timing of delivery and the risk of severe perinatal outcomes, providing additional help in the decision-making process, but it should not be used as a single criterion; it should be used together with other elements, such as the clinical condition of the pregnant patient, the fetus, and others.

## Figures and Tables

**Table 1:** The diagnostic criteria for individual disease entities, in line with the applicable guidelines, were used to qualify the patients for inclusion in the study.

Placental Insufficiency and Guidelines
1. Gestational hypertension, i.e., hypertension diagnosed after 20 weeks’ gestation, with values exceeding 140/90.
2. Preeclampsia, i.e., hypertension with accompanying proteinuria, kidney insufficiency, impaired liver function, visual disorders, thrombocytopenia, swelling, or angiogenic imbalance.
3. HELLP syndrome, i.e., the occurrence of such symptoms as hemolysis, increased aminotransferase concentrations, and low platelet count.
4. Eclampsia, i.e., the occurrence of tonic–clonic seizures secondary to preeclampsia or without preeclampsia.
5. Intrauterine growth restriction, i.e., predicted fetal weight of less than the 3rd percentile for gestational age after ruling out genetic causes (in some of the patients diagnostic amniocentesis, and in others postpartum analyses, were performed).
6. Placental abruption was diagnosed on the basis of clinical symptoms, bleeding, tetanic uterine contraction, with the final diagnosis made postpartum.

**Table 2:** Characteristics of the study group.

Characteristic	Mediana (Min–Max)	Relative Risk (95% CI)
BMI	*n* = 917	29–30
	29 (18–37.5)	
UAPI	*n* = 808	0.9–0.94
	0.915 (0.36–16.7)	
Systolic blood pressure	*n* = 927	138–140
	140 (90–223)	
Diastolic blood pressure	*n* = 927	88–90
	89 (50–140)	
Uric acid, umol/L	*n* = 1011	4.7–4.9
	4.8 (1.73–10.6)	
AST, U/L	*n* = 1028	18–19
	19 (8–883)	
ALT, U/L	*n* = 1027	15–16
	15 (4–967)	
LDH, U/L	*n* = 864	192–198
	195 (90–1748)	
PLT, ×10^9^/L	*n* = 1191	217–226
	221 (41–519)	
RBC, ×10^12^/L	*n* = 1121	4.09–4.15
	4.12 (2.62–8.6)	
Hb, mmol/L	*n* = 1124	7.6–7.7
	7.6 (3.03–9.7)	
Ht, %	*n* = 1191	0.35–0.359
	0.35 (0.24–0.5)	
WBC, ×10^9^/L	*n* = 1123	10.69–10.99
	10.81 (3.9–31.76)	
Fibrynogen, g/L	*n* = 635	4.4–4.6
	4.5 (1.6–33)	
APTT, s	*n* = 1056	27–27.3
	27.2 (9.5–31.76)	
PT, s	*n* = 1050	10.6–10.6
	10.6 (1.1–16.3)	
D-dimers, ng/mL	*n* = 482	1174–1310
	1243 (246.3–13,192)	
sFlt-1, pg/L	*n* = 1127	3558–4021
	3761 (83.6–40,208)	
PlGF, pg/L	*n* = 1127	148–166.6
	86.4 (14.59–994)	
sFlt-1/PlGF ratio	*n* = 1196	25.15–31.63
	28.16 (0.38–1645)	
Delivery week	*n* = 1196	38–38
	38 (18–42)	
Cord blood, pH	*n* = 846	7.31–7.32
	7.32 (6.8–7.49)	

The body mass index (BMI) is the weight in kilograms divided by the square of the height in meters. UAPI—uterine artery pulsatility index; AST—aspartate aminotransferase; ALT—alanine aminotransferase; LDH—lactate dehydrogenase; PLT—platelet count; RBC—red blood cell count; Hb—hemoglobin; Ht—hematocrit; WBC—white blood cell count; APTT—activated partial thromboplastin time; PT—prothrombin time; sFlt-1—soluble fms-like tyrosine kinase-1; PlGF—placental growth factor.

**Table 3:** Correlations between ratio values and perinatal outcomes.

	Ratio_Values vs. Days to Delivery	Ratio_Values vs. Gestational Age	Ratio_Values vs. Birthweight	Ratio_Values vs. Centile	Ratio_Values vs. Ph
Spearman r					
r	−0.4854	−0.3489	−0.3636	−0.1846	−0.1832
95% confidence interval	−0.5288 to −0.4395	−0.3991 to −0.2966	−0.4133 to −0.3117	−0.2405 to −0.1275	−0.2494 to −0.1153
*p* value					
*p* (two-tailed)	<0.0001	<0.0001	<0.0001	<0.0001	<0.0001
Number of XY Pairs	1196	1196	1191	1191	846

**Table 4:** Characteristics of the study group depending on the sFlt-1/PlGF ratio.

		sFlt-1/PlGF Ratio		
	≤40 (*n* = 689)	Relative Risk (95% CI)	>40 (*n* = 507)	Relative Risk (95% CI)
Parity	*n* = 680	1.644–1.788	*n* = 446	1.453–1.615
	1 (1–7)		1 (1–7)	
Gravidity	*n* = 679	1.925–2.114	*n* = 446	1.719–1.936
	2 (1–9)		1 (1–8)	
BMI	*n* = 512	28.76–31.62	*n* = 405	29.82–32.12
	28 (18–37.5)		30 (18–37.5)	
UAPI	*n* = 497	0.71–2.26	*n* = 311	0.7349–2.068
	0.9 (0.36–16.7)		0.95 (0.51–10.6)	
Systolic blood pressure	*n* = 542	132.5–135.7	*n* = 385	147.2–151.6
	130 (90–196)		147 (100–223)	
Diastolic blood pressure	*n* = 542	82.11–84.59	*n* =385	92.5–95.35
	82 (50–140)		95 (55–131)	
sFlt-1/PlGF ratio	*n* = 689	12.5–14.11	*n* = 507	160.3–199.9
	9.92 (0.38–39.96)		101.3 (40.01–1645)	
Delivery week	*n* = 689	37.45–37.83	*n* = 507	35.33–35.99
	38 (20–42)		37 (18–41)	
Birthweight (g)	*n* = 685	2903–3007	*n* = 506	2333–2489
	3000 (300–5120)		2500 (300–4690)	
Birthweight centile	*n* = 685	39.98–44.42	*n* = 506	30.57–35.45
	38.01 (0–100)		24.67 (0–100)	
Cord blood, pH	*n* = 480	7.3–7.32	*n* = 366	7.282–7.3
	7.32 (6.89–7.49)		7.30 (6.8–7.46)	

The quantitative variables are shown as the median (min–max). The body mass index (BMI) is the weight in kilograms divided by the square of the height in meters.

## Data Availability

The original contributions presented in this study are included in the article. Further inquiries can be directed to the corresponding author.

## References

[1] Poon L.C., Shennan A., Hyett J.A., Kapur A., Hadar E., Divakar H., McAuliffe F., da Silva Costa F., von Dadelszen P., McIntyre H.D. (2019). The International Federation of Gynecology and Obstetrics (FIGO) initiative on pre-eclampsia: A pragmatic guide for first-trimester screening and prevention. Int. J. Gynaecol. Obstet..

[2] Bartal M.F., Sibai B.M. (2024). Preeclampsia. StatPearls.

[3] Dimitriadis E., Rolnik D.L., Zhou W., Estrada-Gutierrez G., Koga K., Francisco R.P.V., Whitehead C., Hyett J., da Silva Costa F., Nicolaides K. (2023). Pre-eclampsia. Nat. Rev. Dis. Prim..

[4] Herraiz I., Simón E., Gómez-Arriaga P.I., Martínez-Moratalla J.M., García-Burguillo A., López Jiménez E.A., Galindo A. (2015). Angio-genesis-related biomarkers (sFlt-1/PLGF) in the prediction and diagnosis of placental dysfunction: An approach for clinical integration. Int. J. Mol. Sci..

[5] Verlohren S., Stepan H., Dechend R. (2012). Angiogenic growth factors in the diagnosis and prediction of pre-eclampsia. Clin. Sci..

[6] Herraiz I., Llurba E., Verlohren S., Galindo A., Spanish Group for the Study of Angiogenic Markers in Preeclampsia (2018). Update on the Diagnosis and Prognosis of Preeclampsia with the Aid of the sFlt-1/PlGF Ratio in Singleton Pregnancies. Fetal Diagn. Ther..

[7] Stepan H., Herraiz I., Schlembach D., Verlohren S., Brennecke S., Chantraine F., Klein E., Lapaire O., Llurba E., Ramoni A. (2015). Implementation of the sFlt-1/PlGF ratio for prediction and diagnosis of pre-eclampsia in singleton pregnancy: Implications for clinical practice. Ultrasound Obstet. Gynecol..

[8] Hund M., Allegranza D., Schoedl M., Dilba P., Verhagen-Kamerbeek W., Stepan H. (2014). Multicenter prospective clinical study to evaluate the prediction of short-term outcome in pregnant women with suspected preeclampsia (PROGNOSIS): Study protocol. BMC Pregnancy Childbirth.

[9] Zeisler H., Llurba E., Chantraine F., Vatish M., Staff A.C., Sennström M., Olovsson M., Brennecke S.P., Stepan H., Allegranza D. (2016). Predictive Value of the sFlt-1:PlGF Ratio in Women with Suspected Preeclampsia. N. Engl. J. Med..

[10] Rybak-Krzyszkowska M., Staniczek J., Kondracka A., Bogusławska J., Kwiatkowski S., Góra T., Strus M., Górczewski W. (2023). From Biomarkers to the Molecular Mechanism of Preeclampsia—A Comprehensive Literature Review. Int. J. Mol. Sci..

[11] Thangaratinam S., Allotey J., Marlin N., Dodds J., Cheong-See F., von Dadelszen P., Ganzevoort W., Akkermans J., Kerry S., Mol B.W. (2017). Prediction of complications in early-onset pre-eclampsia (PREP): Development and external multinational validation of prognostic models. BMC Med..

[12] Ukah U.V., Payne B., Karjalainen H., Kortelainen E., Seed P.T., Conti-Ramsden F.I., Cao V., Laivuori H., Hutcheon J., Chappell L. (2019). Temporal and external validation of the fullPIERS model for the prediction of adverse maternal outcomes in women with pre-eclampsia. Pregnancy Hypertens..

[13] Kondracka A., Jaszczuk I., Koczkodaj D., Kondracki B., Frąszczak K., Oniszczuk A., Rybak-Krzyszkowska M., Staniczek J., Filip A., Kwaśniewska A. (2022). Analysis of Circulating C19MC MicroRNA as an Early Marker of Hypertension and Preeclampsia in Pregnant Patients: A Systematic Review. J. Clin. Med..

[14] Peguero A., Fernandez-Blanco L., Mazarico E., Benitez L., Gonzalez A., Boada D., Borràs C., Youssef L., Crispi F., Hernandez S. (2023). Prediction of adverse neonatal outcome at admission for early-onset preeclampsia with severe features. Pregnancy Hypertens..

[15] Binder J., Palmrich P., Kalafat E., Haberl C., Schirwani N., Pateisky P., Khalil A. (2023). Longitudinal assessment of angiogenic markers in prediction of adverse outcome in women with confirmed pre-eclampsia. Ultrasound Obstet. Gynecol..

[16] Graupner O., Karge A., Flechsenhar S., Seiler A., Haller B., Ortiz J.U., Lobmaier S.M., Axt-Fliedner R., Enzensberger C., Abel K. (2020). Role of sFlt-1/PlGF ratio and feto-maternal Doppler for the prediction of adverse perinatal outcome in late-onset pre-eclampsia. Arch. Gynecol. Obstet..

[17] Karge A., Seiler A., Flechsenhar S., Haller B., Ortiz J.U., Lobmaier S.M., Axt-Fliedner R., Enzensberger C., Abel K., Kuschel B. (2021). Prediction of adverse perinatal outcome and the mean time until delivery in twin pregnancies with suspected pre-eclampsia using sFlt-1/PIGF ratio. Pregnancy Hypertens..

[18] Wang L., Mo Y., Wang P., Shen W., Xu L., Zhao G., Lu J. (2024). Prediction Model of Adverse Pregnancy Outcome in Pre-Eclampsia Based on Logistic Regression and Random Forest Algorithm. Altern. Ther. Health Med..

[19] Bednarek-Jędrzejek M., Kwiatkowski S., Ksel-Hryciów J., Tousty P., Nurek K., Kwiatkowska E., Cymbaluk-Płoska A., Torbé A. (2019). The sFlt-1/PlGF ratio values within the <38, 38–85 and> 85 brackets as compared to perinatal outcomes. J. Perinat. Med..

[20] Thadhani R., Lemoine E., Rana S., Costantine M.M., Calsavara V.F., Boggess K., Wylie B.J., Simas T.A.M., Louis J.M., Espinoza J. (2022). Circulating Angiogenic Factor Levels in Hypertensive Disorders of Pregnancy. NEJM Evid..

[21] A Magee L., Brown M.A., Hall D.R., Gupte S., Hennessy A., Karumanchi S.A., Kenny L.C., McCarthy F., Myers J., Poon L.C. (2022). The 2021 International Society for the Study of Hypertension in Pregnancy classification, diagnosis & management recommendations for international practice. Pregnancy Hypertens..

[22] Palmrich P., Binder C., Zeisler H., Kroyer B., Pateisky P., Binder J. (2022). Awareness of obstetricians for long-term risks in women with a history of preeclampsia or HELLP syndrome. Arch. Gynecol. Obstet..

[23] Dijkhuis T.E., Bloem F., Kusters L.A., Roos S.M., Gordijn S.J., Holvast F., Prins J.R. (2020). Investigating the current knowledge and needs concerning a follow-up for long-term cardiovascular risks in Dutch women with a preeclampsia history: A qualitative study. BMC Pregnancy Childbirth.

[24] Yang Y., Le Ray I., Zhu J., Zhang J., Hua J., Reilly M. (2021). Preeclampsia Prevalence, Risk Factors, and Pregnancy Outcomes in Sweden and China. JAMA Netw. Open.

[25] Kumar N., Das V., Agarwal A., Agrawal S. (2023). Correlation of sFlt/PlGF ratio with severity of preeclampsia in an Indian population. AJOG Glob. Rep..

[26] Hughes R.C.E., Phillips I., Florkowski C.M., Gullam J. (2023). The predictive value of the sFlt-1/PlGF ratio in suspected preeclampsia in a New Zealand population: A prospective cohort study. Aust. N. Z. J. Obstet. Gynaecol..

[27] Parchem J.G., Brock C.O., Chen H.Y., Kalluri R., Barton J.R., Sibai B.M. (2020). Placental Growth Factor and the Risk of Adverse Neonatal and Maternal Outcomes. Obstet. Gynecol..

[28] Duhig K.E., Myers J., Seed P.T., Sparkes J., Lowe J., Hunter R.M., Shennan A.H., Chappell L.C., Bahl R., Bambridge G. (2019). Placental growth factor testing to assess women with suspected pre-eclampsia: A multicentre, pragmatic, stepped-wedge cluster-randomised controlled trial. Lancet.

[29] Agrawal S., Shinar S., Cerdeira A.S., Redman C., Vatish M. (2019). Predictive Performance of PlGF (Placental Growth Factor) for Screening Preeclampsia in Asymptomatic Women: A Systematic Review and Meta-Analysis. Hypertension.

[30] Stepan H., Hund M., Gencay M., Denk B., Dinkel C., Kaminski W., Wieloch P., Semus B., Meloth T., Dröge L.-A. (2016). A comparison of the diagnostic utility of the sFlt-1/PlGF ratio versus PlGF alone for the detection of preeclampsia/HELLP syndrome. Hypertens. Pregnancy.

[31] Kwiatkowski S., Bednarek-Jędrzejek M., Ksel J., Tousty P., Kwiatkowska E., Cymbaluk A., Rzepka R., Chudecka-Głaz A., Dołęgowska B., Torbè A. (2018). sFlt-1/PlGF and Doppler ultrasound parameters in SGA pregnancies with confirmed neonatal birth weight below 10th percentile. Pregnancy Hypertens..

[32] Chang Y.S., Chen C.N., Jeng S.F., Su Y.N., Chen C.Y., Chou H.C., Tsao P.N., Hsieh W.S. (2017). The sFlt-1/PlGF ratio as a predictor for poor pregnancy and neonatal outcomes. Pediatr. Neonatol..

[33] Dröge L.A., Perschel F.H., Stütz N., Gafron A., Frank L., Busjahn A., Henrich W., Verlohren S. (2020). Prediction of Preeclampsia-Related Adverse Outcomes With the sFlt-1 (Soluble fms-Like Tyrosine Kinase 1)/PlGF (Placental Growth Factor)-Ratio in the Clinical Routine. Hypertension.

[34] Musilova I., Kremlacek J., Pavlikova L., Holeckova M., Volnerova M., Jacobsson B., Kacerovsky M. (2024). sFlt-1/PlGF ratio is associated with delivery within 7 days in women with spontaneous preterm labor. Am. J. Obstet. Gynecol..

